# Thymoquinone Suppresses Angiogenesis in DEN-Induced Hepatocellular Carcinoma by Targeting miR-1-3p

**DOI:** 10.3390/ijms232415904

**Published:** 2022-12-14

**Authors:** Samer A. Tadros, Yasmin M. Attia, Nadine W. Maurice, Sally A. Fahim, Fatma M. Abdelwahed, Samar Ibrahim, Osama A. Badary

**Affiliations:** 1 Department of Biochemistry, Faculty of Pharmacy, October University for Modern Sciences and Arts (MSA), 26 July Mehwar Road Intersection with Wahat Road, 6th of October City P.O. Box 12451, Egypt; 2 Pharmacology Unit, Cancer Biology Department, National Cancer Institute, Cairo University, Kasr Al Eini Street, Fom El Khalig, Cairo P.O. Box 11796, Egypt; 3 Department of Biochemistry, Faculty of Pharmacy, Cairo University, Kasr El-Aini Street, Cairo P.O. Box 11562, Egypt; 4 Department of Biochemistry, School of Pharmacy, Newgiza University (NGU), Newgiza, Km 22 Cairo-Alexandria Desert Road, Giza P.O. Box 12577, Egypt; 5 Medical Biochemistry and Molecular Biology Unit, Cancer Biology Department, National Cancer Institute, Cairo University, Kasr Al Eini Street, Fom El Khalig, Cairo P.O. Box 11796, Egypt; 6 Clinical Pharmacy Practice Department, Ahram Canadian University, 4th Industrial Zone, Banks Complex, 6th of October City P.O. Box 12451, Egypt; 7 Clinical Pharmacy Practice Department, Faculty of Pharmacy, The British University in Egypt (BUE), Cairo P.O. Box 12508, Egypt; 8 Clinical Pharmacy Department, Faculty of Pharmacy, Ain Shams University, Cairo P.O. Box 11566, Egypt

**Keywords:** thymoquinone, diethylnitrosamine, miR-1-3p, angiogenesis, TIMP3, liver cancer

## Abstract

Hepatocellular carcinoma (HCC) is characterized by its high vascularity and metastasis. Thymoquinone (TQ), the main bio-active constituent of *Nigella sativa*, has shown anticancer and hepatoprotective effects. TQ’s anticancer effect is mediated through miRNA regulation. miR-1-3p plays a significant role in various cancers but its role in HCC invasiveness remains poorly understood. Bio-informatics analysis predicted that the 3′-UTR of TIMP3 is a target for miR-1-3p; Rats were equally divided into four groups: Group 1, the negative control; Group 2 received TQ; Group 3 received DEN; and Group 4 received DEN after pretreatment with TQ. The expression of TIMP3, MMP2, MMP9, and VEGF in rats’ liver was determined immunohistochemically. RT-qPCR was used to measure the miR-1-3p level in rats’ liver, and TIMP3, MMP2, MMP9, and VEGF in the HepG2 cells after being transfected with miR-1-3p mimic or inhibitor; In rats pretreated with TQ, a decreased expression of MMP2, MMP9 and VEGF, and increased expression levels of TIMP3 and miR-1-3p were detected. Treating the HepG2 cells with miR-1-3p mimic led to the upregulation of TIMP3 and downregulation of MMP2, MMP9, and VEGF, and showed a significant delay in wound healing; These results suggested that the anti-angiogenic effect of TQ in HCC may be mediated through the regulation of miR-1-3p.

## 1. Introduction

The prevalence of and deaths from various cancers are rapidly escalating and thus imposing a continuous strain on healthcare systems worldwide. One of the most commonly diagnosed cancers is primary liver cancer, which is also the third leading cause of cancer mortality globally in 2020. In Egypt, liver cancer is the leading cause of cancer death [1]. Hepatocellular carcinoma (HCC) continues to be a significant health burden and accounts for 75–85% of primary liver cancer cases. The main risk factor for HCC is cirrhosis brought on by either the chronic hepatitis B virus (HBV) or hepatitis C virus (HCV). Smoking, excessive alcohol consumption, obesity, and type 2 diabetes have also been associated with an increased risk of HCC [1,2]. In Egypt, the most prevalent cause is HCV infection [1].

HCC patients are asymptomatic prior to diagnosis and more than 70% of HCC cases are diagnosed at late stages, where HCC resection would not be indicated [3]. The limited available treatments for advanced HCC focus more on extending overall survival than they do on curing the condition. These include anti-angiogenic treatment, immunological check-point therapy, radio-embolization, and chemotherapy [4]. Anti-angiogenic therapy is the suggested course of treatment for advanced stages of HCC because HCC is characterized by its high vascularity, which plays a major role in tumor growth and metastasis. This therapy is intended to destroy the abnormally structured blood vessels which causes tumor hypoxia and shrinkage [3,5]. Previous studies have demonstrated highly expressed angiogenic factors in HCC such as VEGF which was highly expressed in about 91% of advanced HCCs [6]. A poorer prognosis in HCC has also been linked to higher VEGFR-2 levels [7].

The efficiency of various traditional cancer therapy platforms is limited by the probability of recurrence and unfavorable side effects from nonspecific toxicity, and insufficient selectivity [8,9]. Consequently, several research studies have been conducted to apply new treatment approaches by using phytochemicals in adjuvant combinational therapy to enhance drug efficacy and diminish drug toxicity [10,11]. Thymoquinone (TQ), the pre-eminent component of *Nigella sativa* black seeds, suggests a promising natural approach in various therapeutic areas, notably cancer [12]. TQ has demonstrated significant anticancer results against various cancer types such as breast [13], bone [14], pancreatic [15], lung [16], and liver cancers [17]. Recently, our study showed that TQ inhibited the initial phase of diethylnitrosamine (DEN)-induced HCC in rats [18]. DEN, a genotoxic substance frequently used to simulate the development of liver tumors in rodents, affects the early stages of carcinogenesis most likely by causing oxidative stress. This damages DNA and cell membranes, causing liver damage, along with an increase in the production of dangerous free radicals. Pretreatment with TQ was proven to protect against hepatic injury and carcinogenesis by reducing oxidative stress and lipid peroxidation, triggering the activity of antioxidant enzymes, and potentiating the apoptotic pathway [19].

The pathological origin of cancer has also been demonstrated to be directly related to the dysregulation of miRNAs. Deregulated miRNA expression in cancer is one of the main characteristics of the disease, affecting the proliferation, angiogenesis, and metastasis of tumors [20]. Previous studies have concluded that miR-301a-3p, miR-448, miR-506, miR-1306-3p, miR-776, and miR-122 have a significant role in HCC via regulating tumor proliferation, invasion, and metastasis [21,22,23,24,25,26]. Moreover, the capacity of TQ to impede tumor progression was found to be correlated to miRNA regulation. It was previously demonstrated that the application of TQ reduced oxidative stress, inhibited necrosis, and potentiated hepatocyte regeneration in mice with Ehrlich acid solid tumors by downregulating the miR-206b-3p expression level [27]. By the same token, using TQ in combination with doxorubicin induced apoptosis through upregulating miR-16 and miR-375 expression in HCC cells [28].

It has been identified that miR-1-3p had a significant role in the occurrence and progression of multiple tumors. Low miR-1-3p expression levels were found in prostate cancer, oral squamous cell carcinoma, bladder cancer, lung cancer, and colorectal carcinoma [29,30,31,32,33]. In liver, miR-1-3p served as an important early diagnostic biomarker for hepatocellular injury [34]. Furthermore, miR-1-3p serum levels were associated with HCC patients’ survival time [35]. It was recently proven that miR-1-3p overexpression prevented the growth of HCC by downregulating SOX9. Further studies are needed to explore the function and role of miR-1-3p in the invasiveness of HCC [36].

This study aimed to investigate the effect of TQ on miR-1-3p expression in HCC and to further understand the role of the TQ/miRNA axis on the angiogenic signaling pathway attributing to cancer occurrence and progression.

## 2. Results

### 2.1. TQ Decreases the Angiogenesis in DEN-Intoxicated Rats

**Figure 1:**
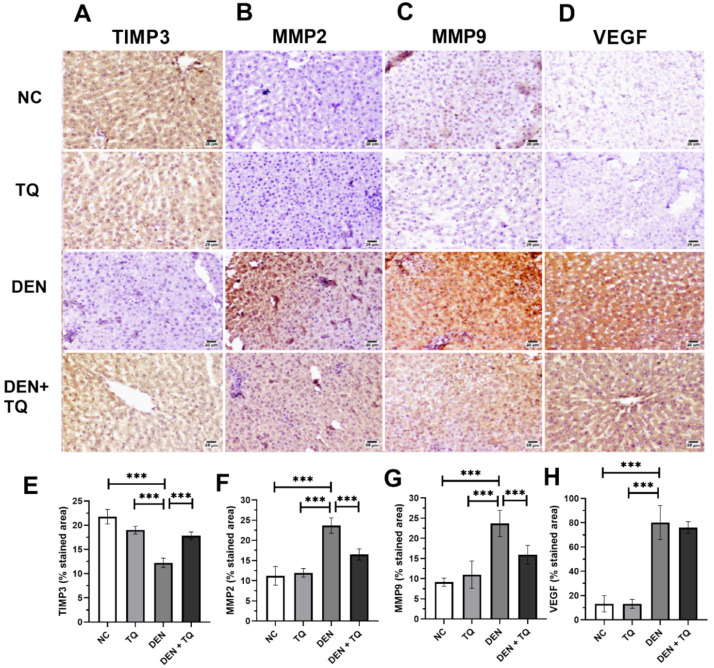
Immunohistochemical staining of rat liver for TIMP3 (**A**), MMP2 (**B**), MMP9 (**C**), and VEGF (**D**). ImageJ analysis and quantitation were done using % of stained area of TIMP3 (**E**), MMP2 (**F**), MMP9 (**G**), and VEGF (**H**), and illustrated by bar charts. Counterstaining was done with hematoxylin and the scale bar equals 25 µm. One-way ANOVA and Tukey HSD as a post hoc test were used to compare the four groups *** significant at *p* < 0.0001. The experiments were performed in triplicates of 6 rats per each group and data are presented as mean ± standard deviation.

The role of TQ in DEN-induced carcinogenesis was confirmed in our previous paper by histopathology, liver function tests and the measurement of HCC biomarkers [18]. To know the status of the angiogenic biomarkers, liver tissues were sectioned for immunohistochemical staining to examine TIMP3, MMP2, MMP9, and VEGF (Figure 1A–D). Our result clearly illustrated a normal expression in the hepatic parenchyma of the negative control (NC) and TQ groups. A high expression of MMP2, MMP9, and VEGF was obvious in DEN-intoxicated rats but the expression of TIMP3 was lowered compared to the negative control and TQ groups (*p* < 0.0001). In rats pretreated with TQ, a decreased expression status of MMP2, MMP9 and VEGF, and increased expression of TIMP3 were obvious (Figure 1E–H).

### 2.2. MiR-1-3p Level Increased in TQ-Treated Rats

**Figure 2:**
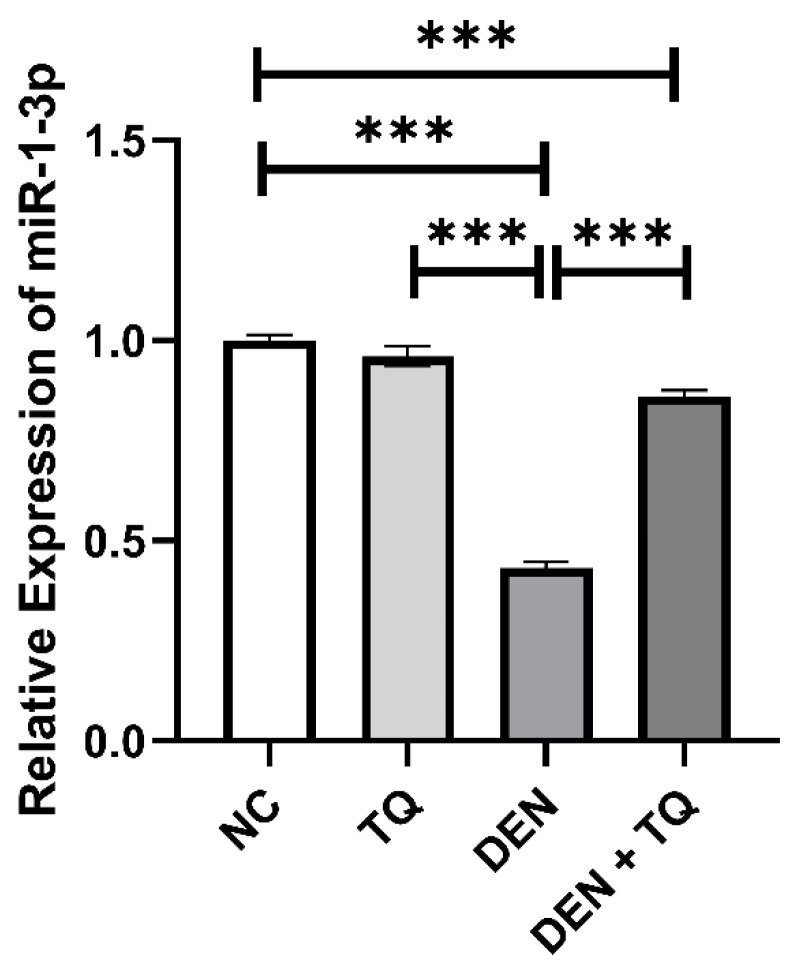
Effect of TQ, DEN, and their combination on miR-1-3p expression in rats’ liver tissue. Pretreatment with TQ restored the miR-1-3p levels compared to the DEN-intoxicated rats. Data are presented as bar charts (*n* = 6). *** significant at *p* < 0.0001.

The effect of TQ on miR-1-3p was explored by measuring its expression in rat liver tissue. The results of qRT-PCR analysis revealed significant downregulation of miR-1-3p in DEN-intoxicated rats compared to the negative control group. Moreover, pretreatment of rats with TQ prominently upregulated miR-1-3p compared to the DEN-injected group (Figure 2).

### 2.3. In Vitro Effect of miR-1-3p on Retardation of HepG2 Cell Migration

**Figure 3:**
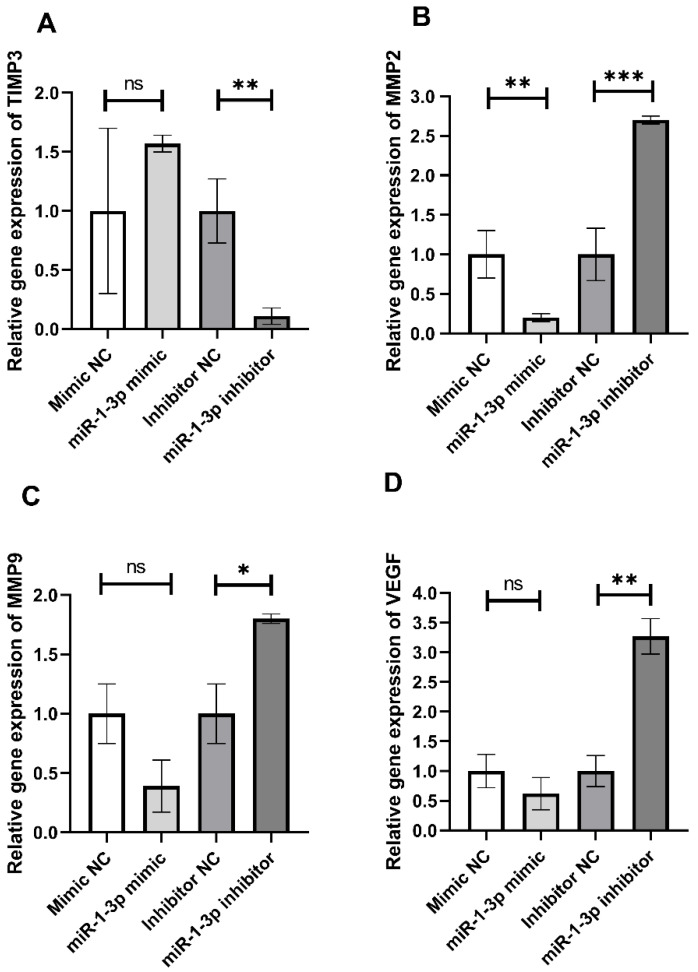
Relative TIMP3 (**A**), MMP2 (**B**), MMP9 (**C**), and VEGF (**D**) mRNA expression in HepG2 cells transfected with miR-1-3p mimic, inhibitor, and their NC. Transfection of HepG2 cells was done with 50 nM of human miR-1-3p mimic, inhibitor, or their corresponding negative controls using HiPerFect transfection reagent for 48 h. Each column represents mean ± SD of three separate experiments. Unpaired *t*-test was used to compare between the two groups. * Significant at *p* < 0.05, ** significant at *p* < 0.001, and *** significant at *p* < 0.0001.

To confirm that the antimigratory effect of TQ is regulated by miR-1-3p, HepG2 cells were treated with miR-1-3p mimic or inhibitor or their corresponding controls. Then, the gene expression levels of the angiogenesis biomarkers, TIMP3, MMP2, MMP9, and VEGF, were measured (Figure 3). Treating cells with miR-1-3p mimic led to a non-significant upregulation of TIMP3 (*p* = 0.23); however, MMP2, MMP9. and VEGF were downregulated (*p* < 0.001 *, MMP2; *p* = 0.14, MMP9; *p* = 0.3, VEGF) when compared to the mimic negative control. On the other hand, cells treated with miR-1-3p inhibitor showed a significant increase in the expression levels of MMP2, MMP9, and VEGF at *p* < 0.0001, 0.01, and 0.001, respectively. On the contrary, inhibiting miR-1-3p decreased the TIMP3 level at *p* < 0.001 when compared to its inhibitor negative control.

**Figure 4:**
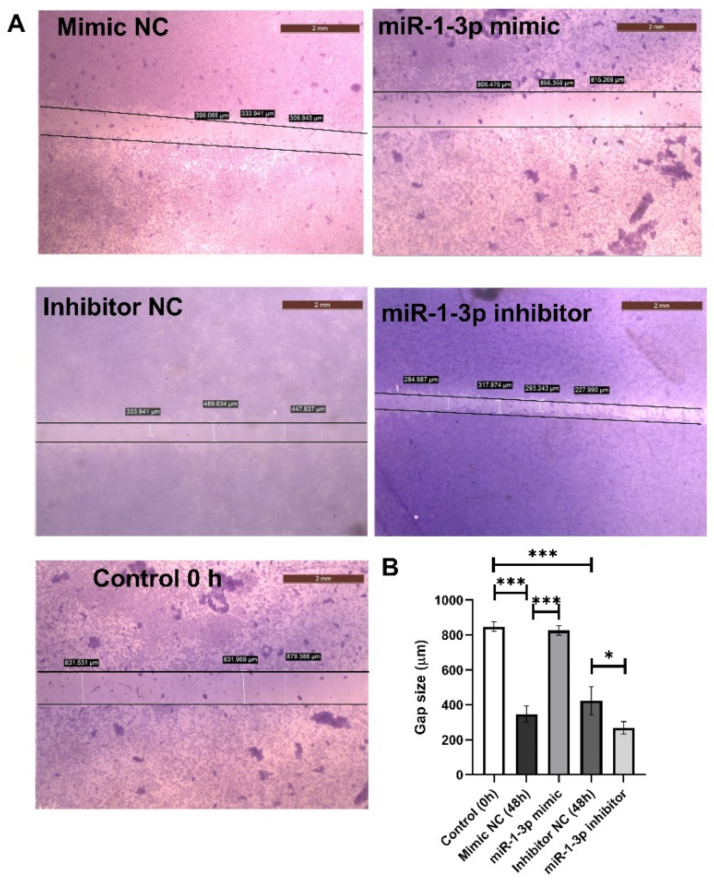
Effect of miR-1-3p mimic and inhibitor on cell migration in HepG2 cells. Representative images showing migrated HepG2 cells following transfection with miR-1-3p mimic, inhibitor, and their NC (**A**). Scratching was done with a 10-µL pipette tip. Quantitative representation of the migration of HepG2 by the wound healing assay (**B**). The data are presented as mean and standard deviation (*n* = 3). The one-way ANOVA test was used to examine statistical differences. Images were taken by an inverted microscope (Scale bar = 2 mm). * Significant at *p* < 0.05 and *** significant at *p* < 0.0001.

To further investigate the effect of miR-1-3p on cell migration, its role in inhibiting HepG2 cell migration was elucidated using an in vitro wound healing assay for 48 h (Figure 4A). The wound in the mimic and inhibitor controls was healed 48 h after scratching a monolayer of cells. HepG2 cells treated with mir-1-3p mimic showed a significant delay in the wound healing of the scratched area compared to its corresponding control at 48 h. The mimic significantly increased the wound gap by 137.6% at *p* < 0.001. On the other hand, treating HepG2 cells with miR-1-3p inhibitor hastened the wound closure, decreasing the gap by 36.6% at *p* < 0.05 when compared to the inhibitor negative control (Figure 4B).

### 2.4. Prediction of Target Genes and Pathway Prediction Analysis for miR-1-3p

The TargetScan and DIANA-TarBase v8 databases, as well as other predictive algorithms, confirmed that the 3′-UTR of TIMP3 is a target for miR-1-3p. The results of these analyses showed that miR-1-3p had two 7mer seed sites at the positions of 1440–1447 and 1756–1762 of the TIMP3 3′-UTR, and aligned with this site with an miRDB score of 91 and context score percentile of 98. Moreover, the results of the search in the miRCancer database showed that miR-1-3p is downregulated in HCC, breast, and bladder cancers. TIMP3 was found to be an inhibitor to MMP2, MMP9, and VEGF in the angiogenesis signaling pathway (KEGG pathway; hsa05205). Finally, as illustrated in Table 1, one hundred and fourteen miR-1-3p-targeted genes with the highest prediction scores were detected using DAVID tools. By using KEGG pathway analysis, these genes were found to significantly regulate various signaling pathways involved in angiogenesis, such as Rap1, tight junctions (TJs), Hippo, proteoglycans, and Ras signaling pathways (*p* < 0.001).

## 3. Discussion

In the present study, the function and role of TQ in inhibiting cell migration in vivo and in vitro were explored via the regulation of miR-1-3p. Moreover, the function and role of miR-1-3p in the invasiveness of HCC by targeting the TIMP3/MMP2/MMP9/VEGF pathway were also determined.

TQ, a phytochemical compound found in natural products, can inhibit cancer cell growth and the spread of cancer cells. Thymoquinone inhibits prostatic cancer angiogenesis and proliferation by suppressing the AKT/ERK pathway [37], and through the NF-κB pathway in osteosarcoma [38]. However, the role of thymoquinone in inhibiting angiogenesis through the TIMP3/MMP2/MMP9/VEGF pathway has not been previously investigated. The immunohistochemistry results of the current study clearly illustrated a high expression of the angiogenesis biomarkers MMP2, MMP9, and VEGF in DEN-intoxicated rats, but the expression of TIMP3 was lowered compared to the negative control and TQ groups. In contrast, rats pretreated with TQ demonstrated a decreased expression status of MMP2, MMP9, and VEGF, and increased expression of TIMP3. Therefore, the result suggests that TQ can inhibit HCC metastasis.

The capacity of TQ to impede tumors’ progression was found to be correlated to miRNA regulation [12]. It has been identified that miR-1-3p had a significant role in the occurrence and progression of a variety of tumor cells and low miR-1-3p expression levels were found in prostate cancer, oral squamous cell carcinoma, bladder cancer, lung cancer, and colorectal carcinoma [29,30,31,32,33]. In liver, miR-1-3p served as an important early diagnostic biomarker for hepatocellular injury and was associated with HCC patients’ survival time [34,35]. Recent research has demonstrated that the overexpression of miR-1-3p, which causes SOX9 to be downregulated, prevents HCC from proliferating [36]. Additionally, miR-1-3p inhibited the growth of colorectal cancer and gastric cancer cells through controlling YWHAZ-mediated EMT [39,40]. In this study, we investigated the effects of TQ on the expression of liver miR-1-3p in DEN-induced HCC in mice. Our study revealed significant downregulation of miR-1-3p in DEN-intoxicated rats compared to the negative control and TQ groups. Pretreatment of rats with TQ prominently upregulated miR-1-3p compared to the DEN-injected group.

To investigate the possible signaling pathway by which miR-1-3p moderates the TIMP3/MMP2/MMP9/VEGF signaling pathway, bio-informatics analysis predicted that TIMP3 is a direct target of miR-1-3p. In addition, TIMP3 was also found to be an inhibitor to MMP2, MMP9, and VEGF in the angiogenesis signaling pathway (KEGG pathway; hsa05205).

MMPs play a critical role in cancer invasiveness and metastasis by regulating the process of epithelial–mesenchymal transition (EMT) [41]. MMP-2 is the major proteolytic enzyme among the MMPs that is expressed in HCC cells but is not often detected in liver cells [42]. In the EMT process, HIF-1, a recognized promoter of HCC invasiveness, downregulates E-cadherin and upregulates MMP-2 [43]. Moreover, MMP-9 cleaves a number of chemokines and activates IL-1 β, which controls angiogenesis and endothelial stem cells [44]. Previous research has shown that highly invasive HCC cells such as SK-Hep-1 and HCCLM3 are more likely to express MMP-2/9 than are weakly invasive and non-invasive cells, such as HepG2 and normal liver cells, respectively [45].

TIMPs are endogenous inhibitors of MMPs, so any disequilibrium between the activities of MMPs and TIMPs may have an impact on the invasion and metastasis of cancer cells. TIMP3 regulates cell death, angiogenesis, and tumor cell invasion [46]. Moreover, restoring TIMP-3 levels induces colon cancer cell death and limits cell proliferation [47]. Loss of TIMP-3 resulted in worse outcomes for oral cancer patients and increased metastasis [48]. TIMP-3 can prevent umbilical vein endothelial cell proliferation and migration by decreasing the expression levels of VEGF-mediated MMP-2 and MMP-9 [49]. Melanoma and lymphoma cells in TIMP-3-/- mice had a higher metastatic ability, MMP-2 and MMP-9 expression levels [50].

**Figure 5:**
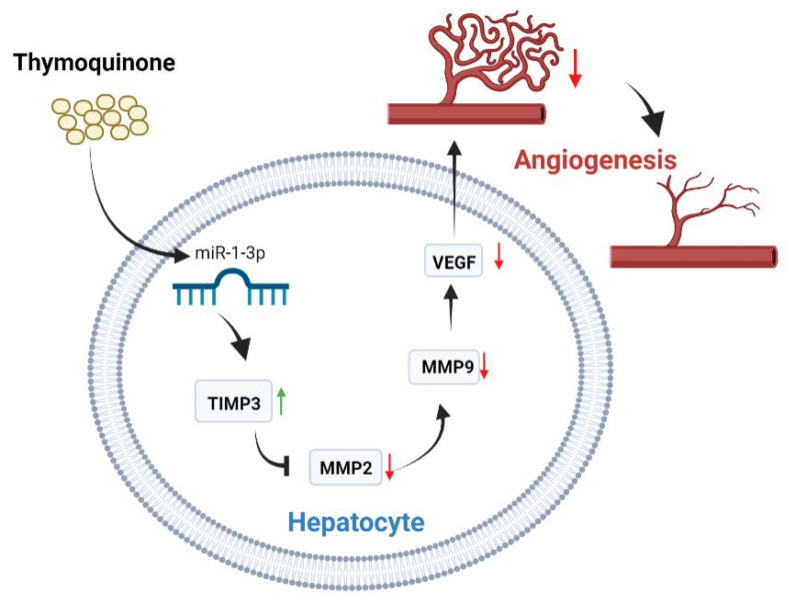
The role of thymoquinone in inhibiting angiogenesis through TIMP3/MMP2/MMP9/VEGF pathway.

To further confirm the anti-angiogenic effect of miR-1-3p, HepG2 cells were treated with miR-1-3p mimic or inhibitor or their corresponding controls. Then, the expression levels of the angiogenesis biomarkers, TIMP3, MMP2, MMP9, and VEGF were measured. Treating the cells with miR-1-3p mimic led to a non-significant upregulation of TIMP3; however, MMP2, MMP9, and VEGF were downregulated when compared to the mimic negative control. On the other hand, cells treated with miR-1-3p inhibitor showed a significant increase in the expression levels of MMP2, MMP9, and VEGF, with a significantly decreasing TIMP3 level when compared to its inhibitor negative control. Moreover, inhibition of miR-1-3p led to an increase in the cell migratory effect in the wound healing assay. Cell migration is essential to angiogenesis and is considered one of the main underlying mechanisms of angiogenesis [44]. The suggested mechanism by which TQ inhibits angiogenesis is illustrated in Figure 5.

Predictive databases were utilized to find the miR-1-3p candidate target genes with the highest prediction scores. The Rap1, TJs, Hippo, proteoglycans, and Ras signaling pathways were all strongly impacted by the miR-1-3p target genes, according to the KEGG database. These pathways were found to be correlated with HCC proliferation and angiogenesis. TJ proteins, through integral proteins, maintain the intactness of epithelial and endothelial cells. They can also regulate the expression of various genes essential for the spread of cancer cells through adapter proteins such as zonula occludens (ZO) [51]. The increase in ZO levels decreased HepG2 cell growth by trapping the cells in the G1 phase of the cell cycle [52]. Similarly, the reduction of ZO protein expression have promoted tumor development in a DEN-induced HCC mouse model, showing that ZO protein plays an important role in HCC progression [53]. Disruption of TJs results in increasing vascular permeability, initiating angiogenesis and further metastasis of tumorigenic cells [54]. A decrease in the expression of TJ proteins such as claudin-5 and claudin-7 was found to be linked to a poor HCC prognosis [55,56].

The Hippo signaling pathway is involved in hepatocyte proliferation, vascular remodeling, migration, and HCC formation through inhibiting the transcriptional factor yes-associated protein (YAP)/transcriptional co-activator with PDZ-binding motif (TAZ) [57]. In addition, the essential elements of VEGF signaling in angiogenesis include YAP/TAZ [52,58]. Proteoglycans have diverse effects on various molecular mechanisms related to the progression of HCC. Proteoglycan 4 was reported to inhibit carcinoma neovascularization, while glypican-3 and agrin increase the proliferation and angiogenesis of HCC cells [59]. The angiogenic and tumorigenic effects of agrin in HCC are mediated through triggering YAP/TAZ activation, and subsequently stabilizing endothelial VEGFR2 [60,61].

The family of Ras oncogenes stimulates the initiation of tumor growth and angiogenesis, where the oncogenic H- and K-Ras stimulate VEGF synthesis and subsequently promote tumor vascularization and development [62]. Increased levels of Ras effectors were associated with a short survival in human HCC patients [63]. Additionally, the Ras/Raf/MEK/ERK pathway, which is crucial in the pathophysiology of HCC, is activated by EGF, VEGF, and platelet-derived growth factor [64,65].

Ras-associated protein 1 (Rap1), a small G protein, is fundamental for angiogenesis. Rap1 positively modulates VEGFR2 activation and thus promotes VEGF-mediated angiogenesis [66]. It is debatable whether Rap genes are tumor suppressors or oncogenes. Rap1 acts as a tumor suppressor and inhibits HEP3B cells’ proliferation. In addition, Rap1-deficient female mice are shown to be more vulnerable to DEN-induced liver injury and HCC [67]. In contrast, the increased Rap1b expression by miR-101-3p suppression enhanced HCC in various liver cancer cell lines [68].

## 4. Materials and Methods

### 4.1. Chemicals

TQ and DEN were purchased from Sigma-Aldrich Corp. (St. Louis, MO, USA). TQ was freshly made by combining it with water (5 mg/kg/day). DEN is dissolved in normal saline at a dose of 200 mg/kg. Detection kits were provided by Biodiagnostic (Giza, Egypt) and the primers were obtained from Bioline Inc. (Taunton, MA, USA).

### 4.2. DEN Liver Injury Model

**Figure 6:**
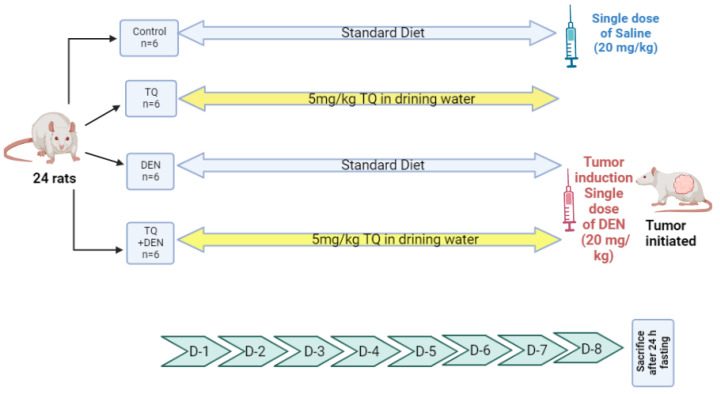
Experimental design. Twenty-four rats were divided into 4 groups (*n* = 6). Group 1 (negative control group): the rats had a regular diet and received saline ip on the 8th day. Group 2 (TQ group): Received TQ for 7 days at a dose of 5 mg/kg/day in their drinking water. Group 3 (DEN control): received a standard diet for 7 days. Group 4 (DEN + TQ): Received TQ (5 mg/kg/day) in their drinking water for 7 days. Both group 3 & 4 had DEN ip on the 8th day at a dose of 200 mg/kg.

DEN-induced HCC in rats’ liver was conducted in twenty-four adult male rats (150–170 g) that were purchased from the animal house at Ahram Canadian University and each group of three was kept together in a cage for a week before starting the study, in order for them to adapt. Then, the rats were randomly divided into four groups as mentioned before in our previous study [18]: (a) control group; (b) TQ group; (c) DEN control group; (d) DEN + TQ group, as shown in Figure 6, and according to the guidelines of the Ethical Committee of Ahram Canadian University (P0221). After 24 h of fasting, the rats were sacrificed by decapitation. The livers of each group were dissected, washed with normal saline, and used for miRNA analysis and immunohistochemical examination.

### 4.3. Immunohistochemistry

Immunohistochemical staining was used to determine the protein expression of TIMP3, MMP2, MMP9, and VEGF in liver tissue samples. Sections of 4 um thickness were impeded in paraffin, fixed, and rehydrated by submersion in xylene and ethanol. The peroxidase activity was stopped by incubating the sections with 3% H_2_O_2_ for 10 min. Heating, then cooling, was done for antigen retrieval. The sections were incubated for 24 h with anti-TIMP3 (cat.no. sc-6836; dilution1:200), MMP2 (cat.no. sc-10736; dilution1:100), MMP9 (cat.no. sc-10737; dilution1:100), and VEGF (cat.no. sc-7269; dilution1:500) antibody purchased from Santa Cruz Biotechnology, Santa Cruz, CA, USA. Next, the sections were incubated with biotin-coupled secondary antibody, then avidin–biotin–peroxidase complex (Vector Laboratories, Newark, CA, USA). Diaminobenzidine (DAB) chromophore (D5367; Sigma, St. Louis, MO, USA) was used to enhance the enzymatic reaction. Counterstaining was done with hematoxylin on an automated linear stainer and coverslipped. A Zeiss ImagerZ1 upright microscope equipped with an AxioCam camera (Zeiss, Berlin, Germany) was used to take images. The percentage of the stained area was measured by using ImageJ processing software after applying the ImageJ threshold filters.

### 4.4. Cell Lines

The American Type Culture Collection (ATCC, MN, USA) provided the HepG2 (liver) cells utilized in our work. In order to preserve tumor cells, serial subculturing using RPMI-1640 media, 1% penicillin/streptomycin, and 10% fetal bovine serum (FBS) was employed at the National Cancer Institute, Cairo, Egypt. Pre-confluent cells were subcultured and incubated at 37 °C in a humid atmosphere with 5% CO_2_.

### 4.5. Transfecting Cells with Mimic or Inhibitor of miR-1-3p

For the in vitro transfection experiments, HepG2 cell lines (10^5^ cells/well) were seeded in 6-well plates and transfected with 50 nM of human miR-1-3p mimic, inhibitor, or their corresponding negative controls (NC) (Cat# mimic:339173; inhibitor:339121; NC:339173, Qiagen, Hilden, Germany) using a HiPerFect transfection reagent (Cat# 301704, Qiagen, Hilden, Germany) in Opti-MEM Reduced Serum Medium (Thermo Fisher, Waltham, MA, USA) according to the protocol’s guidelines. After 48 h following transfection, the cells were harvested for the in vitro experiments.

### 4.6. RNA Isolation and Gene Expression Levels of miR-1-3p, TIMP3, MMP2, MMP9, and VEGF by RT-qPCR

Total RNA was isolated from either the HepG2 cells transfected with 50 nM control miR, miR-1-3p mimic and inhibitor for 72 h, or rats’ liver tissue using Direct-zol RNA Miniprep Plus (Zymo Research Corp., Irvine, CA, USA, Cat # R2072) following the guidelines. The concentration and purity of the resultant RNA was then detected using a Nanophotometer N50 spectrophotometer (Implen, Vernon Hills, IL, USA). Total RNA was reverse-transcribed using an miRNA cDNA synthesis kit (PrimeScript, Takara, Japan) and cDNA archive kit (Applied Biosystems, Foster City, CA, USA) for miR-1-3p and mRNA, respectively. Reactions were incubated at 37 °C for 1 h, followed by the inactivation of the reaction by incubation at 95 °C for 5 min. For miRNA and mRNA expression, qPCR was performed using a KAPA SYBR FAST qPCR Kit (Kapa Biosystems, Wilmington, MA, USA), using 3 μL of diluted RT product (equivalent to 20 ng) as a template in a 20 μL PCR reaction containing 2X KAPA SYBR FAST qPCR master mix, 10 µM forward primer, 10 µM reverse primer, 50X ROX high/low, and PCR-grade water. The conditions for qRT-PCR were as follows: 95 °C for 3 min, followed by 40 cycles of 95 °C for 3 s, then the annealing temperature specific to each miRNA and mRNA for 30 s, and 70 °C for 30 s. The miR-1-3p and each of the TIMP3, MMP2, MMP9, and VEGF gene expression levels were normalized to that of U6 and GAPDH, respectively. Their relative expression levels were calculated by the 2^−ΔΔCt^ method [69]. All primers used are listed in Table 2.

### 4.7. Wound Healing Assay

The effectiveness of the suppression of cell migration and metastases was evaluated using the wound healing assay. HepG2 cells were seeded onto 6-well plates and allowed to reach 80–90% confluency before being evenly scraped with a 10 μL pipette tip. Before being subjected to either miR-1-3p mimic or inhibitor, or their corresponding NC, the cells were washed with sterile PBS to remove debris. An inverted microscope (DFC290, Leica, Wetzlar, Germany) was used to monitor wound closure both right away (0 h) and 48 h afterwards. All experiments were repeated three times.

### 4.8. Relationship between miR-1-3p and TIMP3 at Post-Transcriptional Level

miRNAs regulate the expression levels of various genes by binding to complementary sequences in the 3′-UTR of mRNAs. Bio-informatics analysis using miRDB (http://mirdb.org/mirdb/index.html, accessed on 1 March 2021), TargetScan (https://www.targetscan.org/, accessed on 22 April 2021), and DIANA-TarBase v8 (https://dianalab.e-ce.uth.gr/html/diana/web/index.php?r=tarbasev8, accessed on 23 April 2021) predicted the binding sites and score between the 3′-UTR of TIMP3 and miR-1-3p. Moreover, the miRCancer database (http://mircancer.ecu.edu/ accessed on 22 April 2021) was used to determine the changes in the expression levels of miR-1-3p in various types of cancers. KEGG pathway analysis was used to detect the TIMP3 angiogenesis signaling pathway. Lastly, using online DAVID tools and KEGG pathway analyses, miR-1-3p was analyzed to identify various target genes and signaling pathways associated with angiogenesis in HCC.

### 4.9. Statistical Analysis

The Kolmogorov–Smirnov and Shapiro–Wilk tests were used to determine the normality of the data (*p* > 0.05), and then parametric tests were used: unpaired *t*-test to compare between two independent groups, or one-way ANOVA followed by Tukey HSD as a post hoc test to compare more than two groups. IBM SPSS Statistics software version 26 (Chicago, IL, USA) and GraphPad Prism Software version 9.1.1 (San Diego, CA, USA) were used.

## 5. Conclusions

In conclusion, we reported in this study that TQ enhances miR-1-3p expression, resulting in a change in TIMP3, MMP2, MMP9, and VEGF levels, which finally inhibits cell migration and angiogenesis. These findings not only highlight the function of miR-1-3p-mediated TIMP3 in the control of cell migration and angiogenesis, but they also raise the possibility that TQ might provide a new and effective anti-angiogenic treatment for HCC patients in the future.

## Figures and Tables

**Table 1:** KEGG pathway enrichment analysis of miR-1-3p.

KEGG Signaling Pathway	Number of Genes (%)	*p*-Value
Rap1 signaling pathway	25 (2.9%)	7.4 × 10^−5^
Tight junction	21 (2.4%)	1.8 × 10^−4^
Hippo signaling pathway	20 (2.3%)	2 × 10^−4^
Proteoglycans in cancer	23 (2.7%)	3.6 × 10^−4^
Ras signaling pathway	25 (2.9%)	4.2 × 10^−4^

**Table 2:** Primer sequences.

Gene	Primer Sequence	Accession Number	Annealing Temp (°C)
TIMP3	F: 5′-TCTGCAACTCCGACATCGT-3′ R: 5′-TTGGTGAAGCCTCGGTACAT-3′	NM_000362.5	59
MMP2	F: 5′-AGACAGTGGATGATGCCTTTGC-3′ R: 5′-GGAGTCCGTCCTTACCGTCAAA-3′	NM_001302510.2	61.2
MMP9	F: 5′- TTCCAAACCTTTGAGGGCGA-3′ R: 5′-CAAAGGCGTCGTCAATCACC-3′	NM_004994.3	59.8
VEGF	F: 5’-TCCTCACACCATTGAAACCA-3’ R: 5’-GATCCTGCCCTGTCTCTCTG-3’	NM_001025366.3	56.9
GAPDH	F: 5′-ACCCACTCCTCCACCTTTGA-3′ R: 5′-CTGTTGCTGTAGCCAAATTCGT-3′	NM_001357943.2	59.7
miR-1-3p	F: 5′-TGGAATGTAAAGAAGT-3′	MIMAT0000416	58
RUN U6B	F: 5′-GGCAGCACATATACTAAAATTGGAA-3′	M14486.1	58
Universal reverse primer	R: 5′-GTGCAGGGTCCGAGGT-3′	N/A	58

## Data Availability

Not applicable.
